# “It Is All About Education, Isn’t It?”: Community Priorities for an Aboriginal and Torres Strait Islander Adolescent Nutrition Program

**DOI:** 10.3390/ijerph23040461

**Published:** 2026-04-03

**Authors:** Renae Earle, Robyn Littlewood, Simone Nalatu, Floyd Leedie, Salifu Yusif, Jacqueline L. Walker

**Affiliations:** 1 School of Human Movement and Nutrition Sciences, The University of Queensland, Brisbane, QLD 4072, Australia; 2 Health and Wellbeing Queensland, Brisbane, QLD 4064, Australia; 3 Goondir Health Services, Dalby, QLD 4405, Australia

**Keywords:** qualitative, Indigenous, adolescent, nutrition, prevention

## Abstract

**Highlights:**

**Public health relevance—How does this work relate to a public health issue?**

- Adolescence is a critical life-stage for health and wellbeing. However, in rural areas, there is insufficient support for adolescent empowerment and good nutrition.
- Investments in effective, co-designed adolescent health programs have the potential to support population health.

**Public health significance—Why is this work of significance to public health?**

- This research identifies priorities for adolescent empowerment and nutrition-related health promotion through the voices of adolescents and their broader community.
- A co-designed empowerment-focused nutrition program is developed, with the potential for implementation within the Aboriginal and Torres Strait Islander community-controlled health setting.

**Public health implications—What are the key implications or messages for practitioners, policy makers, and/or researchers in public health?**

- Findings of this research indicate that adolescent and community health priorities are focused on skill development and locally tailored solutions.
- A program outline with the potential to meet adolescent and community health needs is outlined and ready for piloting in a rural community.

**Abstract:**

Aboriginal and Torres Strait Islander adolescents living in rural communities do not have sufficient access to health promotion services. Community programs that respond to adolescent needs, highlight community strengths, and are locally tailored are needed. Set in Queensland (Australia), this study was cross-sectional and qualitative in design. Using implementation science and Aboriginal and Torres Strait Islander frameworks, this study aimed to identify community priorities for the co-design of a culturally appropriate, empowerment-focused nutrition program with rural Aboriginal and Torres Strait Islander adolescents. Through community yarning, the barriers, enablers, and opportunities for program implementation were explored within an Aboriginal and Torres Strait Islander community-controlled health organization. Ten adolescents, two parents/caregivers, eight healthcare staff, six community leaders, and four Elders participated. Thematic analysis identified six themes that outline community health priorities, contextualization to the local food environment, and the importance of cooking skills for empowerment and involving the family unit. Thematic analysis also explored community preferences for program evaluation. Themes were integrated with other knowledge sources to develop a program outline that is aligned with evidence-based practice and community voice. Implementation of the co-designed program is recommended and will be explored in partnership with the community through future research.

## 1. Introduction

Aboriginal and Torres Strait Islander peoples are custodians of one of the world’s oldest living cultures, and holders of knowledge that have kept communities strong and healthy for over 65,000 years [1,2,3,4]. The disruption of Aboriginal and Torres Strait Islander food systems, and forced reliance on a Western diet, has (amongst other factors) contributed to poorer health outcomes for Aboriginal and Torres Strait Islander peoples. Reducing health inequities in Aboriginal and Torres Strait Islander communities is crucial to a healthier future for all and will support national commitments to Closing the Gap [5,6,7,8].

In Queensland (Australia), poor health that is related to having an unhealthy weight disproportionately impacts Aboriginal and Torres Strait Islander young people. One-third, of whom, live with overweight or obesity [9]. Young people with an unhealthy weight are at an increased risk of mental and physical health conditions and consistently report a lower quality of life [10,11,12,13,14,15,16,17,18,19]. Respiratory and inflammatory conditions, type-2 diabetes, kidney, liver, and coronary artery disease are amongst the chronic conditions that disproportionally impact youth living with an unhealthy weight [10,18,20,21,22,23,24,25,26,27]. The effects of poor health in childhood are not limited to the early years of life [18,24,25,26,27,28,29]. Obesity and its related conditions often persist into adulthood, where they act as precursors to weight-related morbidity and mortality and can have intergenerational impacts [10,18,21,26,28,30,31]. Conversely, healthy habits and good nutrition that are established in adolescence may be carried into adulthood and can exert positive impacts intergenerationally [10].

Aboriginal and Torres Strait Islander adolescents living in rural communities do not have sufficient access to health promotion services [32,33]. Community programs that respond to the unique needs of adolescents and are contextualized to their local environment are needed [34,35,36]. Such programs should consider empowerment as a potentially powerful component of obesity prevention and health promotion, particularly in Aboriginal and Torres Strait Islander communities [37,38]. Empowerment programs have demonstrated the ability to holistically improve individual health and wellbeing while promoting community collectivism [39,40,41]. They are particularly well-suited to addressing health inequity, including with Aboriginal and Torres Strait Islander communities [38,39,40,41,42,43,44,45]. However, the principles of empowerment theory are scarcely utilized in Australia for health promotion purposes, particularly with children and young people [38].

Further, calls to include Aboriginal and Torres Strait Islander communities in the design and delivery of nutrition and health programs are a consistent feature within the peer-reviewed literature [46]. Therefore, this research aimed to partner with a rural Queensland community to:

- Identify community priorities and preferences for the co-design of a culturally appropriate, empowerment-focused nutrition program for Aboriginal and Torres Strait Islander adolescents;
- Explore the potential barriers, enablers, and opportunities for implementation of the co-designed program within the research setting.

## 2. Materials and Methods

### 2.1. Research Design

This study was cross-sectional and qualitative in design. It utilized the Aboriginal and Torres Strait Islander research method of yarning and a qualitative survey to achieve its aims. This research built upon a previous study conducted by the authors [47] and was nested within an Aboriginal and Torres Strait Islander community-controlled social and emotional wellbeing program for adolescents [47].

### 2.2. Research Setting and Partnership

This research was undertaken in a rural Queensland community, in collaboration between the local Aboriginal and Torres Strait Islander Community-Controlled Health Organization (hereafter, ACCHO), The University of Queensland, and Health and Wellbeing Queensland. The research partnership was formally recognized in a collaborative research agreement between all parties. In this agreement, the rights of Aboriginal and Torres Strait Islander peoples to their intellectual and cultural property (both existing and created through the research) were protected and recognized as exclusively owned by Aboriginal and Torres Strait Islander peoples. Through the collaborative research agreement and trusting relationships between the research partners, learnings were shared across agencies to support collective knowledge and build network capacity.

The local ACCHO (run by and employing Aboriginal and Torres Strait Islander peoples) was the setting for this research. In Australia, ACHHOs are community-run primary health services that provide culturally informed care for Aboriginal and Torres Strait Islander peoples. ACCHOs empower communities through services that are controlled and delivered by Aboriginal and Torres Strait Islander peoples; they foster holistic and locally tailored healthcare [48]. This research was conducted within the ACCHO under ACHHO governance, support, and guidance. Through the influence of the ACCHO, this research aligned with the principles of community control and community protocols [49]. The researchers followed community protocols by taking advice from community members about research and engagement processes, respecting Sorry Business and other community events, and seeking permission from community governance structures such as Elders and the ACCHO. Guidance from the local community and ACCHO, along with continuous reflexivity, supported researchers to uphold the Principles of Ethical Research Conduct with Aboriginal and Torres Strait Islander peoples [50]. This included Aboriginal and Torres Strait Islander peoples sharing control over data collection and management processes. For example, local Aboriginal people employed by the ACHHO co-facilitated data collection, contributed significantly to data interpretation, and informed how the findings were disseminated. Hence, local Aboriginal people were exposed to research processes and participated in professional development activities related to research but were not formally trained or directly employed by the project.

The aims of this study were developed iteratively and in partnership with the community. The authors visited the community to explore local interest for research and to determine a potential research scope. During the early engagement phases, the community expressed interest in proposed research about empowerment and obesity prevention. The ACCHO identified an existing community program as an ideal setting within which the research could be conducted. The identified program was an after-school social and emotional wellbeing program for adolescents (12–18 years old) (hereafter, the SEWB program) [51]. The ACCHO and broader community identified opportunities to strengthen the SEWB program with general nutrition-related promotion. Hence, this research focused on the co-design (and implementation planning) of an empowering nutrition program to be situated within the ACCHO’s adolescent SEWB program.

### 2.3. Research Approach, Framework, and Theories

All research is grounded within the philosophical stance of researchers, which provides important context and influences all elements of the research process [52]. In recognition of this, the distinct epistemology, axiology, ontology, methodology, and methods that comprise this research approach are explicitly outlined.

The philosophical stance guiding this research is interpretive in nature [53]. This stance is influenced by the position of the research team about the research topic and participants, which is further described below in Section 2.4. Given this research aimed to understand community perspectives and co-design a health promotion program, the research adopts epistemology and ontology that positions knowledge as deeply embedded in experience, human consciousness, social interaction, and context. Our approach recognized that multiple realities can simultaneously co-exist. Accordingly, we chose methods that allowed the co-existence of differing realities between individuals and participant groups. Reflecting the epistemological and ontological underpinnings of the research approach, the methodology was grounded in Indigenous Standpoint theory [54,55,56,57]. Indigenous Standpoint theory preferences the unique knowledge of Aboriginal and Torres Strait Islander peoples and articulates how Aboriginal and Torres Strait Islander lived experience influences reality and understanding of the world [58]. Indigenous Standpoint theory positions this knowledge as absolute truth and complements our interpretivist research approach. Axiology guiding this research included values related to community control, equity, and cultural safety. These values influenced decision-making related to research methods and directed the team towards decolonial research methodology [54,55,56,57]. How these values were operationalized is further outlined in Section 2.4.

To address the second aim of this research (to explore the potential barriers, enablers, and opportunities for implementation of the co-designed program), an additional framework from implementation science was drawn upon. Implementation science is the study of research translation to practical outcomes and explores the complexities of effective implementation [4,32,59,60,61,62,63]. The integrated Promoting Action on Research Implementation in Health Services (i-PARIHS) was selected as it has been designed for use in health services and utilized in similar research contexts [64,65,66,67,68,69]. Further, it is aligned with the philosophical stance of this research. The i-PARIHS suggests that facilitators/researchers (themselves) and various layers of the research context influence knowledge translation and implementation outcomes. Thus, reflecting the philosophical underpinnings of this research, positioning knowledge as not separate from individuals but deeply rooted in human nature, relationships, and the broader context.

In summary, a variety of frameworks and theories were used to guide this research. Each of which served a purpose in meeting the aims of this research. Indigenous Standpoint and decolonial theories guided the research methodology and were utilized to make decisions about research methods. These theories were utilized in all elements of the research process (from conception, design, analysis, reporting, and dissemination) and supported the research process to remain centered in ethical practice [54,55,56,57]. The i-PARIHS was utilized in post hoc qualitative analysis (after inductive theming) and to position findings in the discussion. This approach ensured all aims of this research were fully met. Throughout, internal coherence was maintained by aligning decisions about research methodology and methods with a clearly articulated philosophical stance, epistemology, ontology, and axiology.

### 2.4. Positionality Statement

In qualitative research, the identities and life experiences of authors play an active role in the research process. The authorship team is a combination of non-Indigenous Australian (RE, JW, RL), Aboriginal (FL), Fijian Australian (SN) and African Australian (SY) researchers. It is acknowledged that the identities of the authors have influenced all elements of this research process.

This research was undertaken as part of the PhD Candidature of the lead author (RE), who is a non-Indigenous woman. She lived and worked within the community in which this research was conducted. Other authors are respected leaders in their respective fields of obesity prevention and Aboriginal and Torres Strait Islander health, and experienced researchers in nutrition, public health, and health services. The team occupies various positions about the research, and those who occupy an ‘outsider’ perspective (particularly non-Indigenous authors) approached this research with respect and humility. Their approach has been centered on a deep sense of responsibility and accountability, acknowledging the pervasive impacts of colonization and recognizing the systemic inequities that colonization has perpetuated. The authors have spent considerable time reflecting on, questioning, and examining the various power dynamics related to academic research, and have taken a deliberate approach to share power, promote self-determination, and be led by Aboriginal and Torres Strait Islander colleagues and communities. Previously successful practical strategies that support a culturally safe research approach were utilized (for example, consulting with Elders and sharing data collection processes with local Aboriginal colleagues) [47].

### 2.5. Participants and Recruitment

Yarning circle participants included adolescents participating in the SEWB program, their parents/guardians, community leaders, Elders, and health service staff. Community leaders, Elders, and health service staff were eligible to participate if they had any degree of connection to the SEWB program (and would therefore be well-placed to advise on the co-design of a program situated in this context). For example, community leaders and health service staff who were involved in the delivery of the SEWB program, and the Elders groups associated with the ACCHO. Participants were recruited through convenience and purposive sampling methods utilizing the existing community engagement mechanisms established by the ACCHO [47]. For example, data collection was facilitated through the usual SEWB program delivery and integrated into existing forums such as Elders groups, community leader forums, and health service staff meetings [47]. All participants provided their voluntary, informed written consent prior to participation. For participants less than 18 years of age (adolescents), parental/guardian consent was also required.

### 2.6. Data Collection

Through yarning circles and a written survey form, qualitative data were collected between May and June 2021. Yarning circles were co-facilitated by the lead researcher and a local Aboriginal colleague [70]. Yarning was guided by a series of open-ended questions (reviewed and approved by Aboriginal members of the ACCHO) that explored topics related to adolescent health and empowerment, barriers and enablers to healthy lifestyles, tools, activities, and information that would support adolescent health, and potential program implementation techniques and evaluation measures [47]. In addition to general discussions, participants suggested ideas for a co-designed nutrition program, which were then discussed as a group. When it appeared that a discussion on a particular topic came to a natural end, and the group agreed that all major issues were explored, the data collectors concluded the discussion and/or moved to a new topic [47]. Data collection activities occurred in homogenous groups at a community wellbeing center that was familiar and comfortable to all participants [47].

In response to a request from the community, parents were offered the opportunity to contribute to the research via a written survey. Parents expressed interest in the research; however, they lacked the time to attend a face-to-face yarning circle. Hence, an open-ended free-text survey tool containing an adapted version of the yarning questions was developed. The survey was checked for appropriateness by a local Aboriginal colleague and distributed to parents for them to complete and then return in their own time.

### 2.7. Data Analysis

Audio recordings were transcribed by hand verbatim and de-identified for analysis. The lead researcher confirmed the accuracy of the transcription and familiarized themselves with the data by re-listening to the recording while simultaneously reading the text. Inductive thematic analysis was performed by the lead author with the assistance of Lumivero QSR NVivo 11 (Denver, CO, USA) [71,72]. Data from yarning circles and parent surveys were analyzed for pertinent themes relevant to the research aim, and text was systematically analyzed for meaningful quotations. Preliminary themes were shared with project partners, key research participants, and ACCHO staff to confirm they were a fair and accurate representation of the yarning circles. The lead author further refined themes by analyzing their alignment with the i-PARIHS domains. Sub-analyses, with respect to the participant groups, were also undertaken.

### 2.8. Program Development

Yarning themes were utilized to develop a draft nutrition program outline, which was further refined through ongoing engagement and iterative consultation. The draft outline was shared with the ACCHO and project partners for feedback and advice. Background information from stakeholders about the resources and logistics of the SEWB program was considered to develop a feasible plan. The lead author spent several weeks embedded in the SEWB program to understand its processes and identify nutrition-related health promotion opportunities. Overall, yarning themes (described in the results of this study), discussion with local stakeholders, examples of best-practice, and the knowledge of authors were integrated to develop an empowering nutrition program outline, designed for delivery within the SEWB program. The research team communicated the themes with stakeholders to demonstrate why/how certain activities were included. Through a process of iterative, informal consultation, a final program outline and core delivery principles were established and agreed upon.

This research was approved by the University of Queensland Human Research Ethics Committee (2020/HE002894) and conducted with the permission of the ACCHO.

## 3. Results

### 3.1. Participant Characteristics

Thirty-four community members contributed to the co-design of an empowering nutrition program. Across five yarning circles, ten adolescents, two parents/caregivers, eight healthcare staff, six community leaders, and four Elders participated. An additional four parents/caregivers contributed to the research via a written survey. Of the total sample, 73.5% (*n* = 25) identified as Aboriginal, 20.5% (*n* = 7) identified as non-Indigenous, and 6% (*n* = 2) did not disclose their cultural identity. Participant characteristics are further described in Table 1. Amongst parents and caregivers, 50% were fathers, 33% were mothers, and 17% were grandmothers. Participating health service staff were from a broad range of roles, including SEWB and clinical services (for example, counselor, Aboriginal health worker, general practitioner, and nurse), administration and management (for example, practice manager and program coordinator). Two health service staff were also parents to adolescents participating in the SEWB program, but they participated in this research as staff.

The research was conducted in a small rural community and was nested within the SEWB program, meaning the sample pool was small. For example, the 10 adolescents participating in this research reflected 33% of all SEWB program participants [47]. The sample was a representative group of key informants who were well-placed to participate in program co-design. Because participant recruitment processes were embedded within existing ACCHO engagement forums, non-participants reflected individuals who were unable to attend these forums due to situational circumstances (for example, due to other life priorities), rather than those who actively opted out of the research.

### 3.2. Qualitative Findings

The yarning circles identified relevant information and key themes to inform the co-design of an empowering nutrition program. Preliminary themes related to program content, delivery mechanisms, and evaluation were refined through further engagement with key participants and stakeholders. The resulting six yarning themes describe barriers to healthy eating for adolescents in a rural community, and the options for solutions to address these barriers. Overall, participants stressed the importance of nutrition education that is practical for the local context and grounded in empowerment and skills development. The six themes are evidenced by verbatim anonymous quotations in the text below and in Supplemental File (S1). Authors have purposively included verbatim quotations from community members in the results to align with ethical Aboriginal and Torres Strait Islander research practices.

#### 3.2.1. Theme 1: The Local Food Environment Influences Adolescent Nutrition and Is Becoming Increasingly Difficult to Navigate

Participants in this research (from all groups) understood the association between their local food environment and community nutrition. While participants did not use terminology such as ‘obesogenic’ and ‘food environment’, many demonstrated a sophisticated understanding of how the accessibility and availability of healthy food influenced adolescent nutrition.

Participants described the food environment in their rural town as containing fewer fast-food chains (such as McDonalds and KFC) but still concentrated with discretionary options. Some participants, particularly adolescents, surmised that their community food environment must be healthier than regional and urban centers, because those places had an abundance of fast-food chains and a variety of food shopping outlets. For example, one community leader expressed the following in relation to an urban center, “They got McDonalds down there! They got all down there! And they’re all fat!”. Adolescents expressed a similar sentiment. When asked about whether an urban center would be more or less healthy compared to their community, they responded, “Less. They got cheesecake shops and everything.” And, “(less healthy) because of Maccas”. Some adult participants, however, did not agree that the absence of major fast-food chains made their community healthier. They noted that local takeaway food outlets sold food of similar nutritional quality to the major chains, and suggested that their community was, indeed, saturated with discretionary choices. For example, one community leader commented, “…Obviously they (adolescents in urban settings) have a lot more access to fast foods and stuff, but, then I would argue that we have the (local takeaway) who does deep fried stuff ….” Many adult participants noted the abundance of discretionary options available in their community and saw this as a barrier to a healthy weight and life. At the same time, the absence of major fast-food chains was still regarded positively. Adult participants believed that food from major fast-food chains was cheaper than their local takeaway shops; therefore, making discretionary choices potentially less accessible in their community compared to urban centers.

In contrast to the abundance of unhealthy takeaway options, adult participants noted the lack of healthy, affordable food available in their community. This was seen as an additional barrier to healthy eating for adolescents and their families. This topic is explored further in theme 2.

Older adult participants (for example, older community leaders and Elders) reflected on how their food environment had changed over time to become increasingly complex and difficult to navigate. The concept of a healthy diet was seen as constantly changing and, therefore, difficult to keep up with. One Elder commented, “…What people call healthy eating these days is a lot different to what we’ve been brought up on”. Elders recalled simpler times, when there were fewer food choices and diet was connected to Country and the land. For example, “We were brought up different. Somethings we did have, somethings we didn’t have.” And, “In our younger days, we were bought up on a property and things like that and what that sort of stuff involved. With ya milk, cows’ milk and all that”. Adult participants generally held the view that adolescents today had access to too many different food choices, which were confusing and ultimately led to unhealthy habits. Navigating the current food landscape was perceived as mystifying; one community leader commented, “There’s a lot of confusing messages when you walk up and down the aisles of the supermarket”. Adult participants suggested a lack of specialized health services in their community meant they had little support in understanding perplexing nutrition information and choosing a healthy diet. Dietitians were seen as a potential resource that could help community members navigate their increasingly complicated food system.

#### 3.2.2. Theme 2: Nutrition Education Is an Important Part of Health Promotion and Should Respond to Local Barriers to Healthy Eating

Across different adult participant groups, similar healthy eating barriers were discussed. Adult participants (excluding parents/caregivers) suggested that nutrition education, as part of a health promotion program, was important but needed to respond to challenges at the community and family/household level. This theme was raised by adult participants (excluding parents/caregivers), who were more likely (than adolescents) to conceptualize nutrition education within its broader environment. Parents/caregivers and adolescents did not comment on the importance of nutrition education responding to local barriers to healthy eating—they explored barriers to healthy eating in a broader sense, but did not connect this to nutrition education.

When discussing the proposed contents of a nutrition program, adult participants encouraged education that was relevant to local food affordability, availability, and preferences. Across all adult participant groups, healthy food in the local supermarket was perceived as more expensive compared to urban centers. There was an overall sentiment that the cost of healthy food did not match the financial resources of local people. One community leader commented, “…The health food market is pricing themselves out of the reach of most people”. Similar comments were made by an Elder in the following: “…You got to have this and you’ve got to have that (to eat healthy). Cost you fifty hundred dollars to put in… To get all the little bits and pieces in… We don’t have that”. Adult participants (excluding parents/caregivers) suggested that nutrition education should be sensitive to the financial position of local people and should avoid recommending expensive foods. This is reflected in the words of a community leader: “I don’t think we should be putting pressure on peoples’ finances by saying that you must eat this stuff”. Adult participants perceived healthy food in their community as lacking in range and variety. They recommended that nutrition education should consider the availability and quality of local healthy foods. This is reflected in the comments of a community leader, “And then it has to cater, though, for what’s here in this place that you can buy”. Finally, adult participants commented on the need for nutrition education to respond to local taste preferences and dietary cultural norms. Participants felt that nutrition education activities would be more successful if they were tailored to all aspects of the local food environment and taste preferences. One staff member commented, “…What foods do kids like eating, and how can they be adapted (to be healthier)? Cause I think that if you can adapt things that people already like eating, then that’s easier”.

Adult participants stressed the importance of tailoring education to local family life, which was perceived as very busy. Across all adult participant groups, the time pressures of modern family life were seen as a barrier to good nutrition. These pressures were associated with having two working parents, which was thought to be promoting a reliance on convenience foods that required little to no preparation. This challenge was perceived as seasonal and heightened when the local agriculture industry was in flux. This is described in the words of a staff member, “…It depends on the time of the year, I think that that really affects the way that people eat. Because like, so now, a lot of adults that we look after are working like 50–60 h because the (local agriculture industry) is so big at the moment. So, a lot don’t have time to cook… They’re working 50–60 h in a five-day period. Both parents are. So, I’m guessing a lot of (healthy eating) that’s probably falling to the wayside and they’re just going for quick stuff”.

Practical examples of program activities that were perceived as adequately responsive to the local context included healthy/budget shopping lists, adaptations to staple dishes to make them healthier, supermarket tours (by a dietitian), information on how to read nutrition information panels, and guidance on local healthy takeaway options. Incorporating these activities within a nutrition program was seen as increasing its degree of fit and usability with recipients and within the local implementation environment.

#### 3.2.3. Theme 3: Nutrition Education Should Focus on Community Health Interests and Empower Adolescents to Make Autonomous Decisions

Participants suggested that nutrition education, as part of a nutrition program, should focus on health issues that were important to the local community. Education related to local health concerns was seen as crucial for aligning with the motivations, values, and beliefs of recipients. Across participant groups, various health issues were raised as important. Chronic disease prevention, sports nutrition, and general healthy eating practices were broadly the topics of interest to the community. Health service staff identified health issues that regularly presented in their patients and suggested that nutrition education should center on these topics. For example, one health service staff commented: “I suspect that nutrition is a problem, so I think that people are eating enough just probably not eating the right things… Iron deficiency is a pretty common thing”. Across participant groups, there was consensus on the health issues of importance. These included bone and heart health, sugar, diabetes and dental issues, portion control, vegetable intake, and nutrition for sports/muscle hypertrophy. For example, one staff member commented, “Diabetes is (an issue). I believe we’ve had a few very young, and a lot more younger people, getting diagnosed with diabetes this year”. Nutrition education that responded to these local health concerns was supported and encouraged by all participant groups.

Discussion about adolescent overweight and obesity elicited mixed responses. For example, one parent commented, “Healthy weight/overweight is a problem for adolescents and teenagers in my community because everyone has all different healthy weight”. Meanwhile, some health service staff did not believe adolescent overweight or obesity was a problem in their community. They commented, “… Obesity doesn’t seem to be a big problem in the adolescents I see”. Regardless of their view on adolescent weight, participants across groups agreed that unhealthy choices were problematic and that education was seen as an appropriate solution. For example, one community leader commented, “… There’s a lot of really unhealthy choices being made and a lot of people seem to, at our age, not have great knowledge around what is healthy food”.

Adult participants, particularly health service staff, strongly suggested that nutrition education should focus on the ‘why’ behind recommendations. They wanted adolescents to understand the diet disease relationship and suggested adolescents should be empowered with the knowledge and skills to make their own decisions about nutrition, as opposed to being told what to do. Participants suggested that an adolescent nutrition program would be more successful if it refrained from an authoritarian approach and, instead, promoted empowerment. Adult participants perceived that this type of education would be well-received, as it aligned with adolescents’ growing independence and promoted the sharing of power. This sentiment is reflected in the following comments of a staff member, “Like, not just go ‘this is good’, ‘this is bad’, ‘eat that’, ‘don’t eat that’. There is actually nothing there to say ‘this is what it does for your body’. Like, ‘this helps your body grow, these kinds of foods’… That would be good, I think, yeah, if they actually understood how food works with your body”.

#### 3.2.4. Theme 4: Cooking Is an Important Life-Skill for Adolescents and an Effective Vehicle for Nutrition Education

Community leaders and health service staff perceived cooking skills as important for adolescent development and overall health. Participants felt that cooking skills were being lost at the intergenerational level, as the busyness of modern family life left little time for any home cooking, let alone time for teaching young people. As such, building adolescents’ cooking skills and knowledge (as part of a nutrition program) was seen as advantageous, compared to their limited exposure to cooking in the home and school environments. This sentiment was reflected most strongly by community leaders and health service staff. For example, one staff member commented, “…Who do you learn to cook from? You learn to cook from your parents or your grandparents. So, like if your parents are both working or really busy and then how are they going to have time for that? And it’s not something that’s really taught at school or anything”. Adolescents supported the proposal to include cooking as part of a nutrition program. However, they did not connect this to their overall health or the intergenerational loss of skills. Their interest in cooking related to the fun, creative side of the activity. They expressed interest in learning the skills to cook their favorite and new foods. For example, adolescents commented, “(I want to) Learn to cook new things.”, “I love cooking!” and “I wanna cook curry!” Parents did not comment on the value of cooking for health promotion.

Cooking was seen as a vehicle to promote nutrition education and community/family togetherness. For example, one staff member felt that cooking was a form of experiential learning, “… If you’re cooking with them, they’ll ask you questions. Like ‘why are we doing this?’, ‘why are we doing that?’ and I think that that’s a really good way to learn”. Adult participants also suggested practical activities related to cooking that they perceived would increase engagement and impact. These included gamifying cooking through a MasterChef-style competition. All adult participant groups felt that this competition would create a sense of pride and achievement amongst adolescents by letting them choose and create their own recipes. Further, a MasterChef competition was seen as an opportunity to promote cooking amongst the family unit and broader community by engaging them in the event. Participants also suggested developing a program cookbook that could be taken home to further promote cooking within households.

#### 3.2.5. Theme 5: Involving Parents Could Have Benefits but May Be Unrealistic

Participants had varying perspectives about the involvement of parents in an adolescent nutrition program. Parents were seen as an important part of adolescent networks and support mechanisms. Involving them was seen as potentially beneficial and represented an opportunity to consolidate nutrition skills/knowledge within the home environment. These benefits were recognized amongst many participants. Failure to involve parents was perceived (by adult participants) as risky, with the potential to create a mismatch between adolescent nutrition knowledge and their home food environment. For example, one staff member described the potential risks of creating household stress, “… You don’t want to have children learning all this stuff and then going home and the moms already got the groceries and then you causing more stress at home”.

Consistently, participants agreed that engaging parents was a significant challenge and that their participation (while beneficial) was unlikely and unrealistic. One staff member commented, “but the problem (you) will have with parents is they don’t turn up”. Health service staff members felt that parental engagement with health services and programs was a “touchy subject”. They speculated that the reason behind the lack of parental engagement was due to shame and the fear of being subject to a deficit narrative. Health service staff commented that parents might feel like “they (health programs) say they’re doing a bad parenting job, not feeding my kid good”. Hence, adult participants suggested that collaboration with parents as part of the program should be done in ways that prevented shame and were strength-based.

Adolescents and parents themselves were mixed when it came to parental involvement in the program. Some saw adolescents as independent and, therefore, believed parents should be involved. For example, one parent commented, “No they (parents) should not be involved in the program because adolescents can do the program themselves”. Others, however, thought there could be a combination of independent and parent-involved sessions. Elders and community leaders echoed this sentiment and suggested that (even though they were unlikely to engage) parents should still be invited to participate in some program sessions. One staff member suggested, “Why don’t you have the kids on their own for a couple of times while they’re getting some basic skills and then get the parents in and get kids to show… Show them what they can do, and parents can pick up from there”.

#### 3.2.6. Theme 6: Metrics Related to Nutrition Behavior and Knowledge Are Important Success Indicators

Adolescents did not comment on how the program should be evaluated and did not voice preferences on what should or should not be measured. Adult participants, however, had varying perceptions about how program success should be defined. Parents felt that weight-related markers, nutrition, and physical activity behaviors were the best way to test the effectiveness of the program. Other adult participants, however, had differing views on weight-related markers. Some staff did not believe they were a good indication of health, particularly amongst a pediatric population. For example, one staff member commented, “…It’s focusing on the wrong thing and because like you can… Weight can negatively affect your health, but it isn’t actually… Like your weight is not necessarily correlated with your nutritional status”. Overall, there was some confusion amongst health service staff about the appropriateness of Body Mass Index (BMI). However, across all adult participant groups, there was consensus that health behaviors were an appropriate measure of health and program success.

Adult participants also suggested metrics related to nutrition knowledge and wellbeing. However, these were not as strongly supported as health behavior measures. For example, one community leader suggested, “…Their understanding of that sugar intake would be one thing that you said. Like if they can relay back to you like “that’s got five spoonfuls of sugar”. Yeah, you know, that they’ve actually taken it on-board”. Additionally, one staff member suggested, “… How do the kids feel? Because I think that’s really important. Because if they are eating really well… If they eating really well throughout this program, but they feel miserable, like that’s no success”.

The Supplemental Files contain further participant quotes relevant to each of the six themes.

### 3.3. Program Co-Design

The qualitative findings of this research were integrated with local knowledge, subject matter expertise, and engagement with Aboriginal and Torres Strait Islander colleagues to generate an empowerment-focused nutrition program (see Supplemental File (S2)). The program outline was designed to be delivered as an adjunct program nested within the SEWB program. In alignment with the needs of the SEWB program, the nutrition program was designed to be delivered on 1–2 days per week (over 45 min–1.5 h sessions) across 8 weeks. Sessions were structured around empowerment activities, didactic nutrition education sessions, and practical application of nutrition knowledge through the development of cooking skills. Program design was tailored to the implementation context, including its setting, resources, duration, and community preferences. Each of the six themes (documented above) has been reflected in the program design. For example, the program has a focus on cooking skills, empowering adolescents with knowledge to make their own decisions about nutrition, and addresses the local barriers to healthy eating by tailoring recipes to what is locally available and affordable. The program has a focus on seven curriculum topics:

- Sugar and diabetes prevention.
- Empowerment.
- Iron and iron deficiency anemia prevention.
- Healthy heart and cardiovascular disease prevention.
- Healthy bones and osteoporosis prevention.
- Vegetables and fiber.
- Healthy food in the community.

In addition to information gathered through this research, the program co-design responded to the peer-reviewed literature about nutrition-related health promotion and insights from the ACCHO about what is likely to be successful in the SEWB program environment. The peer-reviewed literature suggested that including elements of empowerment (and related activities) can confer additional benefits in childhood obesity prevention [38]. Empowerment (as a component of health promotion) was viewed favorably by the ACCHO and aligned with the objectives of the SEWB program. As such, empowerment theory and specific empowerment activities were incorporated in the development of the program, for example, activities related to journey mapping, ambition building, and community events (i.e., the Murri MasterChef competition).

Further informal discussions with Aboriginal health service staff about barriers related to parental engagement helped identify opportunities that were incorporated into the program design. These included:

- Inviting parents to a small number of sessions.
- Setting “home challenge” tasks for adolescents that were intended to spark conversations within the family unit about nutrition.
- Creating a program cookbook for families that could stimulate cooking in the home.

Despite the important role of physical activity in health promotion, local colleagues advised that the co-designed program did not need to include physical activity, as the usual SEWB program delivery contained ample opportunities for physical activity.

Previous research has identified success factors about the SEWB program that are important to the community [47]. Some of these success factors were purposefully amplified in the program co-design, for example, facilitating access to positive role models and incorporating activities to promote career readiness [47]. SEWB program strengths were highlighted by including empowerment activities that are related to community role models, ambition building, and pathways to employment/study in the co-designed program.

The ACCHO reinforced the need to embed sustainability and consider ways for the program to continue after the research project concluded. To support this, a train-the-trainer model was incorporated as part of the program design, and resources were developed for the ACCHO that would support ongoing program delivery (for example, a program handbook). The program name was decided upon by adolescents, who made suggestions and then voted on their preference. The program was named “Cooking Monsters”.

## 4. Discussion

Yarning with 34 participants identified six themes that informed the co-design of an empowerment-focused Aboriginal and Torres Strait Islander adolescent nutrition program—Cooking Monsters. The themes reflect the views of a broad cross-section of community members, including adolescents themselves. Overall, the themes emphasize the importance of health promotion initiatives responding to their local context/recipients and identify opportunities that could strengthen implementation efforts. Themes identified by this study are consistent with previous literature about the nutrition priorities of Aboriginal and Torres Strait Islander peoples. Specifically, strength-based approaches, food accessibility and affordability, nutrition knowledge and cooking skills, and connection to the family unit [73,74,75]. The themes distilled from yarning circles were taken, with insights gathered from other local knowledge sources and the peer-reviewed literature, to co-design a nutrition program with a focus on cooking and food literacy. The program responds directly to the priorities and suggestions of Aboriginal and Torres Strait Islander adolescents and their broader community. Similar programs with Aboriginal and Torres Strait Islander adolescents have demonstrated positive outcomes [76,77].

Adolescents comprised 29% of the total sample (*n* = 10 of 34) and, by sample size, represented the largest participant group (refer to Table 1). However, across themes, the voices of adult participants are more salient. This reflected the reality of yarning sessions, in which adolescents were less likely to actively express their own ideas or thoughts about program co-design. The adolescents responded best when ideas and opinions of others were presented to them for comment. Compared to the contributions by the adults, the adolescents rarely made meaningful connections between the program and its potential outcomes (such as health, wellbeing, and empowerment) or how these outcomes would be evaluated (theme 6). Previous research about the SEWB program, within which this research is nested, has confirmed that fun and overall enjoyment are primary drivers for adolescent participation [47]. This is one potential reason why adolescent contributions to this research are more strongly aligned with topics/themes that they perceived as directly influencing their enjoyment of the program (for example, cooking and a MasterChef competition), and less evident in discussions about less ‘fun’ topics (for example, evaluation and health outcomes). Further, it is possible that a power imbalance between adult data collectors and adolescent participants influenced how adolescents contributed to the research. The research team was mindful of power dynamics and made efforts to reduce their potential impacts by co-facilitating data collection with a trusted local colleague and building relationships with adolescents prior to data collection. However, it remains possible that power played a role in adolescent engagement and influenced their contributions to the research. In future research, a youth-led approach in which young people conduct research with their peers is recommended [78,79,80]. This approach may yield more meaningful and authentic participation from adolescents and effectively center youth voices in research related to them [78,79,80]. In some instances, contradicting perspectives between participants or participant groups arose. For example, in themes 3, 5, and 6, there were mixed responses from participants with respect to the issue of adolescent obesity, parental involvement in the program, and appropriate evaluation metrics (respectively). The purpose of this research is to highlight the various viewpoints of community members participating in this research. In alignment with an interpretative approach, it is acknowledged that multiple truths can co-exist simultaneously. Understanding is rooted in the life experiences and beliefs of individuals, which are varied and unique. As such, it is not unexpected that unique and varied perspectives were raised in yarning circles. This research has taken the approach to honor the multifaceted contribution of research participants, identify points of consensus, and utilize these to co-design a program.

The themes can be positioned within the broader literature related to implementation science, Aboriginal and Torres Strait Islander health, and empowerment theory. The i-PARIHS framework has proved a useful tool in identifying the elements of implementation science that are most important to participants of this research [64,65,66,67]. In this study, the implementation activity is the proposed nutrition program, and yarning circles highlighted program elements and implementation processes that would contribute to its success. The i-PARIHS defines successful implementation as a function of innovation (the program), recipients (adolescents and their families), context (the local setting), and facilitation (how the program is delivered, and by whom) [68,69]. Themes of this research touch on all domains of this framework, but most strongly resonate with the implementation context, innovation, and recipients. In themes 1–2, the local food environment (which is part of the implementation context) is highlighted as critically important. Participants commented that, to be successful, the program must make recommendations that align with what is available, affordable, palatable, and of interest within the local community. In themes 3 and 6, participants emphasized the need to understand and respond to the knowledge, attitudes, beliefs, skills, and motivations of recipients (adolescents and their families). As such, the resultant co-designed program proposed a flexible delivery model with multiple opportunities to engage the family unit. It directly addressed community-identified health priorities and knowledge/skill gaps. Overall, themes identified opportunities for the program to increase its usability and degree of fit within the local context, which are established enablers of successful implementation [68,69]. The role of the local food environment in nutrition was a strong theme (theme 1). Participants viewed their environment as playing an active role in shaping adolescent health. The influence of food environments (and broader food systems) on nutrition and health is well established, and reflects existing public health models such as the social determinates of health and the socioecological model of health [81,82,83]. Insights about the role of fast-food chains/convenience takeaway foods in adolescent health can be positioned as a commercial determinant of health, which are being increasingly recognized in Aboriginal and Torres Strait Islander health frameworks [84]. Aboriginal and Torres Strait Islander conceptualizations of health are holistic and highlight the need for harmony between the environment and the community. Participants of this research discussed the disconnection between their food environment and health and positioned this as negatively influencing adolescent nutrition. Further, the community highlighted the need for a focus on overall adolescent empowerment/confidence; thus, aligning with the principles of Aboriginal and Torres Strait Islander Culturally Appropriate Health Promotion (specifically, principle 2—socio-culturally tailored health promotion techniques) [85].

Understanding the local food system and Aboriginal and Torres Strait Islander community perceptions about food and nutrition supports the development of relevant and feasible initiatives that address the many factors impacting nutrition [3]. Incorporating this into a multifaceted approach is considered most effective [3,33,86]. Despite participants of this study clearly understanding the relationship between their environment and nutrition (themes 1 and 2), nutrition and cooking education (themes 2 and 4) were seen as the priority for health promotion. Participants suggested that adolescents should be armed with nutrition education, empowering adolescents to navigate their food environment (rather than influence it) (theme 2). This suggests that personal choice and education are where participants felt an adolescent nutrition program could exert influence, despite it being well accepted that the determinants of health are often beyond the control of individuals [87,88]. It was not explicitly addressed in this study; however, it is possible that participants felt they had little influence over their food system, and therefore, did not suggest interventions related to this in the co-design process. This would align with research acknowledging that food systems are determined by multisector actors rather than adolescents and their families [82]. However, further research is required to confirm this with participants.

The overall focus of the program on nutrition education and cooking skills strongly reflects one dimension of the psychological empowerment model (intrapersonal empowerment) [89]. Cooking skills and nutrition education target the intrapersonal component of empowerment by increasing perceived control, competence, and self-efficacy. However, psychological empowerment comprises two additional dimensions (interactional and behavioral), which relate to how feelings of intrapersonal empowerment translate to action and can be positioned within the broader context of one’s community and life [89]. This translation of intrapersonal empowerment was discussed as a potential benefit of the program (for example, adolescent cooking skills translating into home cooking and preventing the intergenerational loss of nutrition knowledge amongst families). With the exception of the Murri MasterChef competition (which promotes community cohesion and the behavioral component of psychological empowerment), participants mostly suggested activities that would strengthen the intrapersonal dimension of empowerment. Less emphasis was placed on targeted strategies that would translate skills to broader empowerment and sustainable practice change. Future research that explicitly addresses how intrapersonal empowerment may translate to overall psychological empowerment in this context is encouraged.

### 4.1. Strengths

The Aboriginal and Torres Strait Australian adolescent nutrition program co-designed through this research is unique in that it aligns with empowerment theory and is embedded within a community-controlled SEWB youth empowerment program [38,47,51]. This is important because empowerment is an under-recognized determinant of Aboriginal and Torres Strait Islander health [37] and a key principle of Aboriginal and Torres Strait Islander Culturally Appropriate Health Promotion [85]. Further, most health promotion research in the outside-of-school-hours care (OSHC) setting is focused on children aged 5–12 years. The Cooking Monsters program is novel in that it is tailored for delivery in the OSHC setting during adolescence (a critical time-period for the development of health behaviors) [90,91]. This research, therefore, highlights OSHC as an additional opportunity for health promotion in adolescence. Finally, this research addresses gaps in the existing literature related to adolescent health promotion in the rural Aboriginal and Torres Strait Islander community setting [32,33,34,35,36] and responds directly to community-identified needs. This research goes beyond qualitative exploration and problem definition by working with the community to co-design a practical and bespoke program tailored directly to the needs of rural Aboriginal and Torres Strait Islander adolescents and their community.

In nutrition research with Aboriginal and Torres Strait Islander communities, deficit discourse is pervasive and can have negative impacts [92,93,94]. The findings of this study showcased local strengths by sharing community voices, lived experiences, knowledge, and ideas to co-design a nutrition program that is anticipated to enact change and support better community health. This study leveraged existing community strengths through being situated within, and building on the efforts of, an existing highly valued community-controlled SEWB program [47]. The participants acknowledged the importance of positive framing, strength-based nutrition education, and empowering adolescents to self-determine their health (themes 3 and 5). This emphasis on empowerment supports existing literature, which has identified empowerment as potentially conferring additional benefits in health promotion, particularly with Aboriginal and Torres Strait Islander and pediatric communities [3,38,92].

This research addressed well-documented needs to collaborate and share leadership with Aboriginal and Torres Strait Islander peoples [3,33,95]. It is strengthened by being situated within the community-controlled setting and by upholding principles of ethical research practice [48,49,50]. The research has shared power with Aboriginal and Torres Strait Islander adolescents, their broader community, colleagues, and co-authors by building and maintaining a genuine cross-cultural partnership grounded in reflexivity and respect [96,97]. This research utilized Aboriginal and Torres Strait Islander research methods, and research processes (from conception to dissemination) were co-led by non-Indigenous and Aboriginal colleagues. Together, these factors have contributed to a reciprocal partnership [98].

### 4.2. Limitations

The views expressed by participants of this research may not reflect the broader community, particularly those who do not engage with the ACCHO (as recruitment methods were centered on health service engagement forums). This can be viewed as a limitation, as it is possible that the findings of this study do not represent the views of the whole community. However, participants and the views they expressed were highly relevant to the proposed implementation context and well-suited to co-developing a tailored program. Resultantly, the co-designed program may have limited generalizability to other community settings and/or other rural communities across Australia. However, the overall co-design approach and general themes of this research have the potential to inform future research endeavors of a similar nature in different communities and contexts.

Yarning circles included discussion about potential program evaluation metrics (theme 6). However, participants did not provide detailed insights about their preferences related to these metrics (particularly adolescents). This is of significance because previous reviews have identified evaluation as a limitation in Aboriginal and Torres Strait Islander health promotion programs, and the need to co-design all elements of programs (including their evaluation) with communities is well established [77]. It is recommended that community stakeholders and existing literature about strength-based evaluation be further consulted in the development of program evaluation [99]. Future research should document the process of program implementation and community impacts.

More than half (66%) of parents/caregivers opted to participate in this research via written survey rather than yarning. Advantages of yarning (as compared to a written survey) include being able to ask follow-up questions, facilitate group discussion and consensus, and collaboratively build ideas [70]. It is possible that the insights gathered from parents could have been more meaningful and cohesive if collected in a yarning format. However, parents/caregivers openly expressed time constraints that prevented them from attending in-person yarning sessions and therefore opted to participate in ways that met their needs.

### 4.3. Program Implementation

The empowerment-focused nutrition program co-designed by this study was subsequently piloted and evaluated using mixed-methods [100]. Implementation of the program (in the same setting in which it was designed) yielded positive outcomes related to community acceptability, feasibility, and satisfaction [100]. The results indicated that (despite minimal improvements in quantitative measures) the program was well-received and community stakeholders advocated for its continued implementation. Research partners have continued working together to secure additional funding for future program delivery. This anticipated funding will support program continuation on a short-term basis with the view to building evidence for a sustainable and long-term funding model.

## 5. Conclusions

This study drew upon community voice, empowerment theory, and implementation science to develop a bespoke adolescent nutrition program that was designed specifically for a community-controlled after-school SEWB program. Through yarning circles with adolescents, Elders, community leaders, parents/caregivers, and health service staff, community priorities related to adolescent nutrition and empowerment were identified. The community felt that health promotion should be tailored to the local food environment and local health concerns. Cooking and nutrition education were seen as vehicles through which adolescents could be empowered to self-determine their health. These insights, and additional engagement with the community, were used to co-design a bespoke program tailored specifically to the community. Future research should continue a meaningful partnership with the community, maintain a strength-based approach, and consider sustainable implementation models.

## Figures and Tables

**Table 1:** Participant characteristics (*n* = 34).

	Adolescents (*n* = 10)	Parents/Caregivers (*n* = 6)	Health Service Staff (*n* = 8)	Community Leaders (*n* = 6)	Elders (*n* = 4)
Cultural Identity (%)					
Aboriginal	100%	83%	63%	17%	100%
Non-Indigenous	0%	0%	37%	83%	0%
Did not disclose	0%	17%	0%	0%	0%
Age					
Years (median, range)	13.5 (12, 15)	44 (36, 60)	36 (23, 57)	32 (19, 64)	Not reported ^1^
Gender (%)					
Female	50%	50%	62.5%	50%	75%
Male	50%	50%	37.5%	50%	25%

^1^ *n* = 1 Elder disclosed their age, which is not reported to protect participant anonymity.

## Data Availability

Data are unavailable due to privacy and ethical restrictions. As the data collected in this research are qualitative and difficult to de-identify, the data are not being made publicly available in the interests of protecting participant anonymity.

## Supplementary Materials

The following supporting information can be downloaded at: https://www.mdpi.com/article/10.3390/ijerph23040461/s1, Supplemental File S1: Additional Participant Quotes, Supplemental File S2: Draft Program Outline.

## Abbreviations

The following abbreviations are used in this manuscript:

ACCHO	Aboriginal and Torres Strait Islander Community Controlled Health Organization
SEWB	Social and Emotional Wellbeing
OSHC	Outside of School Hours Care
