# Supplementary material for: “It Is All About Education, Isn’t It?”: Community Priorities for an Aboriginal and Torres Strait Islander Adolescent Nutrition Program
Source: Int J Environ Res Public Health. 2026 Apr 3;23(4):461. doi: 10.3390/ijerph23040461 (PMC13116386; doi:10.3390/ijerph23040461)
Supplement: Supplementary file 1 [file ijerph-23-00461-s001.zip › ijerph-4196453-supplementary.pdf]

Supplemental Files

<u>Supplement S1 Additional Participant Quotes</u>

**Theme**	**Participant Quotes (by participant group)**
Theme 1: The local food system influences adolescent nutrition and is becoming increasingly difficult to navigate.	<u>Adolescents</u><br>"Oh, there's never healthy food."<br>Researcher: "So, you think that that's something that stops you from eating healthy, because it's not around?"<br>"Sometimes."<br>Researcher: "So do you think in (this community) particularly, we're pretty good at healthy lifestyles? Or, how do you think we go?"<br>"We're better than (regional centre)."<br>Researcher: "Better than (regional centre)... Yep, why do you have that…"<br>"Because of Maccas."<br>"Maccss and KFC and all that stuff."<br>"I like KFC though."<br>Researcher: "Yeah, so there's not many fast food stuff around here. But is there is other unhealthy stuff you can get in (this community)? Or what do you usually get?"<br>"There's some pizzas. From the bakery."<br>Researcher: "And what about in comparison to somewhere like (urban centre), big cities? Do you think they are healthier or less healthy?"<br>" Less. They got cheesecake shops and everything."<br>Researcher: "Yeah. So what do you think we can do here (at SEWB program) to make it easier?"<br>"Get rid of all the junk stuff!"
	<u>Community Leaders</u><br>"And I could not even tell you what is regarded as a health food as opposed to what you can buy at the supermarket…"<br>"They got McDonalds down there (in urban cities)! They got all down there! And they're all fat!"<br><br>"Um, when I was there (at the local school) the tuck shop was open every single day, every lunch break, and I never ate at the at the tuckshop purely because there were very minimal healthy options. Um, everything was processed, um, not much like fruit available or salads that kind of thing. So I think that probably has a huge impact on kids that rely on getting their lunch from the tuckshop every day."

	"… And if you go down to the city you certainly have a bigger range. Even to go to (regional centre) Woolworths, you see they're got a bigger range of fruit and a bigger range of vegetables that sometimes we don't get here. But we've got to just understand that we're in a small community and for those storeowners to stock that stuff (is too difficult)."<br>"But, there's more… More variety now 365 days a year. When I grew up, we used to have seasonal fruit and veg… But now, and the supermarket is telling, um, the growing community that the consumer demands everything every day of the year. And so that's making it very difficult for the growers in Australia because, in the time when we can't grow it for a seasonal perspective, it's important."<br>"I think that's really interesting because obviously they (adolescents in urban settings) have a lot more access to fast foods and stuff, but, then I would argue that we have the (local takeaway) who does deep fried stuff , the (local service station) does deep fried stuff, (another local takeaway) does deep-fried."<br>"Mmm, plenty of access!"<br>"So that's already three places in a very small community where you can access fast food and, you know, as well as those places will sell energy drinks and soft drinks, so I don't know. And it's, you know, those kids who are in the biggest places probably have more access to dietitians, you know, healthier options I suppose. So, I think it's much of a muchness isn't it?"<br>"I don't know why we're getting fatter. Is it because we've got more options of fast food?"<br>"It's the lifestyle. It's just the access has changed and like you know 20-30 years ago you, might have, out here, had one place that you could get deep fried food. Whereas now, it's everywhere."
	<u>Elders</u><br>"What I just noticed though is what people call healthy eating these days is a lot different to what we've been brought up on. What we used to eating."<br>"(there are many foods) That we didn't grow up eating, and now, to turn around and learn cooking with all of these things to eat healthy. It's a bit very different. Yeah, you know a lot of people just don't like that stuff and just don't use it, simple as that."<br>"Sometimes you go cooking with them, it's different… it tasted alright. We, I, got up on, red beans, red kidney beans. That's what they got in the chilli beans - chilli what's the name there. (I was) Never brought up on that."<br>"We were brought up different. Something we did have somethings we didn't have."

	"In our younger days, we were bought up on a property and things like that, and what that sort of stuff involved. With ya' milk, cows milk and all that."<br>"yeah well that's what you had."
	<u>Health Service Staff</u><br>"If we have chocolates in the fridge or biscuits in the cupboard and the fruit bowls on the table, I choose the chocolates. So… less… having less chocolates in my house and more fruit, it's better for me."<br>"I don't think it (overweight) does (have as big of an impact in the community), because we have less fast food here. You know we have the (local takeaway) we have (another local takeaway), and we have um…. Um, and there's (another local takeaway). But then you know so, and there's the (local service station), so those would be your main sources (of unhealthy food) and a lot of that's just like deep fried stuff. In the city they… it's like relatively expensive like $5 or $6 for a sausage roll or whatever it is. Oh, and the bakery as well. Like it… that's relatively expensive if you got a big family whereas yeah in the city there's like 7-11, McDonald's, like all this stuff that's available and I think… And then the same exact same pressures in terms of time exist so a lot of those limitations… the only thing that's probably different is the cost of food."<br>"Yeah and I think, I suspect that obesity rates would probably be higher in places like Brisbane."<br>"…Because they (adolescents in the community) have less access to Maccas and that sort of stuff."
	<u>Parents</u><br>"The biggest factors impacting on adolescent healthy weight and lifestyle in my community are big things. Some things that can prevent adolescents' healthy weight and behaviours would be takeaways and restaurants."<br>"There is not many fast-food places in town. Healthy selections at shops and supermarkets."<br>"Healthy weight is a problem for adolescents and teenagers elsewhere. It is a bigger problem compared to other towns like (regional centre) because they have takeaways and restaurants."<br>"Bigger issue in bigger places."
Theme 2: Nutrition education is an	<u>Community Leaders</u><br>"I think we're really limited out here too, like when… I know I do most of my groceries away because we don't have a big health food section out here in our grocery stores and the limited stuff we do have is very expensive to buy out here."

important part of obesity prevention and should respond to local barriers to healthy eating.	"There's a lot of confusing messages when you walk up and down the aisles of the supermarket. I buy apple juice with no added sugar and then I notice the other day that they've got the same brand right next door to it which says 20% less sugar… Which is better? What does that mean? If you've got no... But already got no sugar, how can you have 20% less than none? Just doesn't make sense!"<br>"Plus, we have a very low socio… And obviously (urban settings) would have their low socio-economic communities. But I just think generally across the board, our community, just statistically is you know, not very wealthy. Um, and that obviously affects your choices around food and also, activities, you know, what the kids can do. And then, that can impact on education levels of parents and kids and all of it kind of builds in together to create some challenges."<br>"I notice that, yeah, even in the supermarket here, like frozen meals seem to disappear fairly quickly. Like pre-packaged stuff that's really fast to make. And I know everyone's sort of time poor but if you, um, yeah I guess it's just that comment about if that's what you're eating when you're in the home, it's, um, some sort of influences your choices when you leave too."<br>"But it almost seems to me like the health food market is pricing themselves out of the reach of most people. And I think if you go into (local supermarket) there's plenty of fruit and veg there to give you the range of what you need to… To eat healthily. Yeah, I don't think we have to do anything special and I don't think we should be putting pressure on peoples' finances by saying that you must eat this stuff."<br>"(Regarding package frozen vegetables) So, cut the top off the plastic, tip her in, and away you go. So, there is no knowledge, no knives, there is no peeling, so it goes on and on and on. So, I think it if that's one way to get people to eat them, that's pretty simple to me."
	Elders<br>"And then they tell you, you got to have this, and you've got to have that. Cost you fifty hundred dollars to put in, to get all the little bits and pieces in… We don't have that."<br>"I think veggies and fruit are deer! They're deer!"<br>"It's very hard not to get something that hasn't got sugar in it"<br>"Very… Yeah yeah. It takes a fair bit to stay on top of that stuff hey."<br>"I don't even read 'em me self, so."<br>"Me either."<br>"I buy what I can afford."

	"Because we diabetes so we got to go and buy certain foods and it's too dear.
	<u>Health Service Staff</u><br>"And it's probably worse now because we are living at a faster pace so working parents… both parents are working now so when they go home, they are looking for that easy option, whether its pasta and meat, or pasta and hot chips or whatever. Or a chook on bread like…"<br>"Something quick and easy"<br>Researcher: "Is there anything else we think (is a barrier to healthy eating in the community)?"<br>"I think the price"<br>"And the quality of the food and what we actually do get out here."<br>"I think as well like it depends on the time of the year, I think that that really affects the way that people eat. Because like, so now, a lot of adults that we look after are working like 50-60 hours because the (local agriculture industry) is so big at the moment. So, a lot don't have time to cook. I don't know, I have multiple patients barely… They're working 50-60 hours in a five-day period. Both parents are. So, I'm guessing a lot of (healthy eating) that's probably falling to the wayside and they're just going for quick stuff. Whereas there is probably other times where… where it's a bit easier."<br>"'Cause I do think it's a lack of knowledge, you know. And alright… And then it has to cater, though, for what's here in this place that you can buy. Because I know that if it's easier to get, you know you gonna get it."<br>"…Like it's important to… Like well not only based on what's accessible here, but like what foods people… What foods do kids like eating and how can they be adapted? 'Cause I think that if you can adapt things that people already like eating, then that's easier."<br>"(Better) Than introducing something that is weird, or just like not like appropriate to this setting"
Theme 3: Nutrition education should centre community health concerns	<u>Adolescents</u><br>"I wanna be fit as well. I want to improve on my fitness as well."<br>"I wanna get fitter."<br>"I want to increase my strength and fitness."<br>"I don't want to be fat."<br>Researcher: "…Do you think that healthy eating and exercise and that sort of stuff is important?"<br>*(Nods from around the circle)*

and interests whilst empowering adolescents to make autonomous decisions.	“Yes.”<br>Researcher: “Is it that what you’re eating is making you feel good? Or, is it fitness for sports? What’s an important part of health for you guys?”<br>“Both.”<br>Researcher: “Like, why is that healthy for you?”<br>“Uh, so that you can stay active and not just sit around and gain weight. That sort of stuff.”<br>“How much food is like too much?”<br>*(Adolescent flexing his muscles)*<br>Researcher: “Foods to get big muscles, is that what (Adolescent) is saying?”<br>Co-facilitator: “Yep, (Adolescent’s) saying they wants foods to get big muscles.”<br>Co-facilitator: “Yep, I’d like to know about all that stuff. Diabetes.”<br>“Me too!”
	Community Leaders<br>“But there’s a lot of really unhealthy choices being made and a lot of people seem to, at our age, not have great knowledge around what is healthy food and what are good choices. And, I guess that’s because they’ve missed out on that education at another point in their life, so, they get into this adult stage where they are allowed to make their own choices. They are not making very good ones”.<br>“Depending on what phase adolescents have grown up with, in the family home a lot of the fresh fruit and veg may not be palatable. Um and I guess that’s where, call it junk food for want of a better word, um, it is palatable because it is all full of sugar and or whatever it might be. And I… you know that’s a hell of a hurdle…”<br>“I mean maybe just on that fresh and frozen stuff. Like yeah, that the nutritional value of something just because it’s processed slightly differently or presented differently doesn’t mean that it’s not as good.”<br>“And I think if we can get the kids to understand what they’re looking at with the labels... So that they’re… They’re looking for the no added sugar because some of those fruit drinks are just like soft drinks really without the bubbles.”<br>“You know, you have that, okay, when you go to the supermarket you go to the fruit and veg and get as much stuff from there to fill up your trolley. You know that’s three quarters of your trolley and then the other one quarter is processed stuff, some dairy…You know that kind of visual easy stuff that it’s kind of a hard and fast rule to help people navigate through all the tiny labels and stuff.”<br><br>“It’s not easy… I’ve done a bit of education on nutrition stuff and I still go to the

	supermarket and just… If I'm tired and can't be bothered, then I just look at the star rating as opposed to fully reading the label."<br>"Give them some guidelines or something to, I mean if you put some… Some goals up there or even some information that they probably never even thought of before. Like, just have a look at the things on the supermarket shelf and see what's in them before we just grab it. And, that one next door might be a better option than the one that you've picked. Yeah, so it's just.. I hate the word, but it is all about education isn't it?"
	Elders<br>"Yeah, like you're saying that energy tables everything on the back."<br>"I mean there's even sugar in barbecue sauce and everything."<br>"There is sugar in everything."<br>"Yeah everything"
	Health Service Staff<br>"It doesn't look like obesity is as a big'a problem. I suspect that nutrition is a problem, so I think that people are eating enough just probably not eating the right things. But obesity doesn't seem to be a big problem in the adolescents I see. Iron deficiency is a pretty common thing."<br>"Yeah, I haven't actually seen a lot… Like statistics-wise with obesity or overweight in adolescents. But, lots of iron deficiency because they not eating enough meat or veggies and most of them because they… Like when mums, like, were pregnant with them, mum was iron deficiency so mainly due to that… So, it could be both female or male but common in female because of that, yeah… Yeah, normally mum deficient, iron deficient, and then they will be born with iron deficiency, never corrected or replaced, and then still they nutrition is not that… not meat, veggies that much, yes."<br>"…It's like 'here eat fruit, it's good for you'. Yeah, but like what does it actually do? Like you eat more of this food for your iron, but how does that help your body? Like, not just go 'this is good', 'this is bad', 'eat that', 'don't eat that'. There is actually nothing there to say, 'this is what it does for your body', like 'this helps your body grow, these kinds of foods'. Um, you know 'this will help with your bones', 'this will help with your…'. But you know… Yeah that would be good, I think, yeah if they actually understood how food works with your body."<br>"…When we have kids with iron deficiency, like it's just not enough. Like, you can say, like, one or two sentences. But it's a lot of information, it's a lot of things happening is... You know. You can explain stuff but you've only got so much time

	(in a medical consult).”<br>“You gotta talk on the level… to make them understand.”<br>“(I want adolescents to know) Why is iron different in this source, this source, this source, this source… So, iron deficiency, I think, is a big issue here but it’s like why? Like why? How things interact? Why… why is iron important? Like, how do you get it from one food and not from another? Like, how does one food prevent the absorption of another?”<br>“Diabetes is (a problem). I believe we’ve had a few very young, and a lot more younger people, getting diagnosed with diabetes this year. From when I went and then come back, I was like Holly Molley! So that would be good, and I think definitely bone.”<br>“Dental health and sugar is just like… And I don’t know about adolescents ’cause again, like, they just they just don’t come in. But like, the amount of kids I see with like black teeth. Like, sometimes their whole mouth is black and you’re just like… Like, ‘argh’. And I don’t, I mean they, they usually will go get them pulled out and then their adult teeth will come and then maybe they start brushing or whatever. I don’t think that’s unique to here, I think that’s an everywhere problem. But I don’t… I don’t know if that’s an issue for adolescents but it’s definitely an issue for kids under 10 or 8 or whatever.”<br>“B12 is another thing.”<br>“Yeah, so it would be good, if you could, yeah, give them the skills to just go…”<br>“Like they’re very active. Um, whether they are then getting enough kind of fuel to support that I guess is a bit of a question. But, then you also do see some kids which are probably going a bit the other way and… And looking like they’re, um, you know, maybe heading towards a bit of an unhealthier path. Yeah, and maybe that’s probably a bit concerning as well ’cause it’s a hard place to start from.”<br>“I think whatever you do like they should have ownership of it. It needs to come from their own minds”
	<u>Parents</u><br>“Healthy weight/overweight is a problem for adolescents and teenagers in my community because everyone has all different healthy weight than other adolescents and teenagers.”<br>“Being taught about healthy eating, especially before sport and during sporting activities.”<br>“Our community would benefit from a healthy lifestyle program for adolescents because, so, they don’t get overweight.”

	"It would be important for an adolescent healthy lifestyle program that includes healthy cooking skills, physical activity, healthy food education and boxing activities."<br>"Importance of healthy eating, healthy options, limit screen time, chronic disease."
Theme 4: Cooking is an important life-skill for adolescents and an effective vehicle for nutrition education.	<u>Adolescents</u><br>Researcher: "Okay, what about cooking? do you guys do much cooking at home?"<br>"I love cooking!"<br>Researcher: "What sort of stuff do you like to cook?"<br>"Everything."<br>"I like cooking! I like cooking!"<br><br>Researcher: "Is there anything else you wanna know how to cook?"<br>"I wanna cook Curry."<br>"I want to learn how to cook onions cut onions."<br>"I want to cook... pies… Yea, home-made pies, guys. Not from the freezer."<br>"(I want to) Learn to cook new things."
	<u>Community Leaders</u><br>"I'm thinking is there scope for (the program) to do, like, a healthy Murri eating cookbook or something you know… To come up with something like that."<br><br>"And it'll be easy stuff, but it also gives them a sense of pride and achievement if they have an actual book that they've taken home that they've made themselves and they know how to cook the stuff in that book. Like I reckon that's really great idea."<br><br>"Well some of the kids don't get that (cooking skills) at home! That's what it's all about."<br>"Its that confidence of cooking."
	<u>Elders</u><br>"Sometimes you go cooking with them, it's different… it tasted alright. We, I, got up on, red beans, red kidney beans. That's what they got in the chilli beans - chilli what's the name there. (I was) Never brought up on that."
	<u>Health Service Staff</u><br>"But again, if parents don't have time, if kids and parents don't have cooking skills. And sometimes, like, I've seen… Where the whole family like, grandparents, kids… Grandparents, parents and kids… Like, all three generations don't know how… Like, have no cooking skills or have never lived in a place where like food is a regularly available thing."

	"Yeah there's nothing that sort of guides them in cooking and what to eat."<br>"Um yeah, but if they had like a few recipes or something for breakfast which you know the kids could actually do themselves and that would make their parents go 'oh, oh righti-o they can do that themselves'."<br>"And like if you think about it, like who do you learn to cook from? You learn to cook from your parents or your grandparents. So, like if your parents are both working, or really busy, and then how are they going to have time for that? And, it's not something that's really taught at school or anything. And if it is, it's often a lot of what they teach is not stuff that you'd actually cook and it's often quite an expensive subject for kids to do, or at least it was when I was at school."<br><br>"Yeah if you got a house where you've got like multiple families living in the same house. Which, as I understand, it's pretty common here because of housing insecurity. Like, it's usually not realistic to I don't know (learn to cook), yeah."<br>"It's also, like you know, a lot of the parents don't have the skills. Like, that's… Lots of adults can't cook for all the same reasons."<br>"And then that change the cycle again. But, because those kids coming through… So, when they start to have kids, that cycle starts to change."<br>"At least you are like, planting the seed for change. Even though it might not be a thing that will change like with these kids at least you're planting the seed and they like… Oh, and even if they had something, oh well, I remember I learned this it was really easy"<br>"And I'm not saying that we should go up to them and be like 'this is the healthy food, this is why you should eat it'. But, I mean I… It would be great if you got like the dietitian in then like for, uh, uh, blocking up for a couple of hours. Get them in and they're like cooking with the kids and like getting to be like a familiar person… And it's also you know, they might, kids are curious, you'll be… If you're cooking with them, they'll ask you questions. Like, 'why are we doing this?', 'why are we doing that?'. And, I think that that's a really good way to learn and to make kids comfortable with the health care system…"<br>"I've got a suggestion about the… The cooking and that. Why don't you ask all the kids what's their favourite meal, like an easy one… Yeah, then get them to go online 'cause there'll good at, um, technical. Go online find an easy recipe and then they all cook those meals but make a recipe book for them."<br>*(Strong support from yarning circle participants)*<br><br>"Another program for (the program) to run as a one off, annually, could be a Murri

	chef where you challenge other people to come in and have that cook off and they can learn some skills as well while they're doing that. It teaches them what they've learned but also, I don't know, you cook your signature dish. That's alright!"<br>"That's a good idea."<br>"Have the Elders here as judges as a little mini master chef…"<br><br>"But even just to preparing food and that… Like, a few of the activities that the kids help, when (charity organisation) was cooking here and they've done that a couple times and they pulled the kids in here to help prepare and, yeah, they loved it. They love doing the different stuff. Cutting the different things up."
Theme 5: Involving parents in an adolescent obesity prevention program could have benefits and maximise impact but may be unrealistic.	<u>Adolescents</u><br><br>Researcher: "We could have the option of doing some stuff with your parents as well. Who likes that idea or do you want it to be just kids?"<br>"Kids."<br>"I reckon both."<br>"Both"<br>"Both"<br>"Yeah"<br>"I think both."<br>"Both I guess."<br>Researcher: "Like, we do kids and parents together, or we do just kids and then just parents?"<br>"Together."<br>"Separate."<br>"I reckon separately."
	<u>Community Leaders</u><br><br>"I think that you should be concentrating on the parents of those age kids"<br><br>"I was just gonna go on from what you were saying about building that habit. But, a lot of that sort of starts in the home like what your parents cook and what you end up choosing to go and buy."<br><br>"Is there some sort of a chance then that there could actually be some sort of a conflict then, between what they (adolescents) actually want to do, what they're able to do here? And actually, what they want to do at home, but, can't because choices are not there?"

	<u>Elders</u><br><br>Researcher: “Um, but it is really tricky because parents are so busy…”<br>“Yeah, they work.”<br>“They should make time but…”<br>“If it’s for your kids you should make time. If they’re thinking of the welfare of their children and things like that, you make the time, you get the time and you do it.”<br>“That’s my view too.”<br>“Unfortunately your generation and the generation that we’re talking about are completely different.”<br>“Yeah that’s true.”<br>“You got to get them (parents) to come to you! And this is the way to do it here. Bit of tukka, they’ll come in for tukka.”<br><br>“Well to get them here too is one thing… And get them to listen while they’re here is another thing.”
	<u>Health Service Staff</u><br>“I’ve got a suggestion. Why don’t you have the kids on their own for a couple of times while they’re getting some basic skills and then get the parents in and get kids to show… Show them what they can do, and parents can pick up from there? And they won’t sort of feel that they are being judged or anything.”<br><br>“I think we also need, if we’re teaching the kids, I think we need to educate parents too. So parents… Parents can actually buy the foods that they need for the kids or get the food.”<br><br>“So even they are scary words for people. When you hear exercise physiologist, they are like “oh”. Dietitian they are like “oh”.”<br>“They (parents) take it like a personal attack on them.”<br><br>“I think that’s important to include the parents into that sort of thing. Because, if the kids learn and the parents don’t have buy-in, they still won’t… They’re not the ones buying the food or doing the grocery. So, they need that parental buy-in. So, they’re getting that stuff, they can make it at home”<br><br>“Yeah, you don’t want to have children learning all this stuff and then going home and the mom’s already got the groceries and then you causing more stress at home. And then that is just gonna go out the window, ya know…”

	"And if you wouldn't get the parents in you could kind… Kind of sort of work on a budget friendly healthy shopping list. And then, that would relate back to your recipes and then we're not adding too much stress, too much stress for everyone."<br>"And you may have discussed this already, but the problem you will have with parents is they don't turn up."<br><br>"I think the only way we will get them is if, your idea (Staff member). Um, if the kids like cooked the meal for the parents and invited him to a dinner where they provided the meal for the parents and then… yeah. But, they'll be caught once, but they won't be caught again. So, if you were to do it again… Try and to load them with information at the next one… they won't."<br>"And the other thing as well is that if you've got some parents show up and then some parents don't. Like the kids of the parents that don't as well. It's balancing all that."<br><br>"…They may have a fear that well… It's something else… I'm going to go to that they say they're doing a bad parenting job, not feeding my kid good."<br>"Touchy subject"<br>"Yeah, it is."<br>"But in saying that, don't give up on the parents, still invite them to do stuff. But be aware, that don't be disappointed when you invite 20 parents and 2 turn up."
	<u>Parents</u><br>"No they (parents) should not be involved in the program because adolescents can do the program themselves."<br>"(Parents should be involved in) Joint session that are fun, interactive. Individual sessions as well for the information sessions."
Theme 6: Metrics related to nutrition behaviour and knowledge are important success	<u>Community Leaders</u><br>"Maye their understanding of that sugar intake would be one thing that you said. Like if they can relay back to you like "that's got five spoonfuls of sugar". Yeah, you know, that they've actually taken it on-board."<br>Researcher: "And it's not something that we may, probably won't see a change in it, or over the course of a school term or something like that. Um, but that's sort of where our key like health behaviours, intake, activity, anthropometrics like your height and weight. Because it is still important to have those data points to track kids."<br>"100% yeah."

indicators.	"You know, anytime you go the doctors they take your height, weight, whatever blood pressure."
	<u>Health Service Staff</u><br>"I'm not necessarily a fan of using height and weight as markers of health 'cause I don't think they are. Especially BMI. Especially in kids… 'Cause I don't think those are markers. They're not actually markers of health. Um, and I think that it's focusing on the wrong thing and because like you can… Weight… Like, weight can negatively affect your health but it isn't actually… Like, your weight is not necessarily correlated with your nutritional status. You can be overweight and be completely mal-nourished and you can be underweight and fully nourished. Like it doesn't… And, I think that it's not a good marker…"<br>"I completely agree. That was one of my pet hates was BMI. And I know we use it a lot as indicators…. (Explains example of football player with a high BMI)… Yeah so I always, always used him as an example of, example of, why I don't fully agree with BMIs and all that because here is a super fit professional athlete. But off… going of that indicator, he's obese."<br>"I think it's helpful like I mean 'cause the majority of people aren't super athletic, so I think it does have some kind of role."<br><br>"I think it (BMI) has some kind of role in adults. But it's not I think in this age group it I think it especially doesn't have…"<br><br>"It's (BMI) done on Asians.. to be so underweight."<br><br>"It (BMI) doesn't have a role. I don't… the weight is not part of this age group, and I think that measuring the success of a program by weight loss, especially in a time of rapid change and puberty has such wide margins. And also, puberty affects girls younger. Like I just, don't think weight is a good marker in this age group. And I think that… And what is success in regard to weight? Is it gaining is it losing? It's just not… I find it very problematic."<br><br>"And social and emotional wellbeing (is a good measure). I think, like, that confidence, like this, I don't know what measures there are… If there's some kind of survey or something you can do like, how do they, how do the kids feel? Because I think that's really important. Because, if they are eating really well… If they eating really well throughout this program but they feel miserable like that's no success.

	Like it's not success 'cause they're doing the right thing but they're hating it the whole time."
	<u>Parents</u><br><br>"It is important to measure for this program are height, weight, physical activity, dietary and nutrition and I would like these to be measured a lot at our program". "Height, weight, daily food."<br><br>"There is nothing in particular that I would prefer you did not measured in this survey."

<u>Supplement S2 - Draft Program Outline</u>

Program Week	Content
1: Sugar and diabetes	• Dietitian to talk about sugar.<br>• Natural vs processed<br>• Reading sugar on labels<br>• Sugary drinks (teaspoons model)<br>• Relationship between sugar, diabetes and dental health
	• Cooking lesson – a healthy drink (smoothies)<br>• Home Challenge: Read the label for sugar content on two household items.<br>• Home Challenge 2: Brainstorm a name for the program.
2: Empowerment	• Story mapping – past, present and future<br>• Identifying inner strengths<br>• Handling peer pressure – role play<br>• Plan for empowerment project – Murri MasterChef
3: Iron and	• Dietitian to talk about iron (food sources and diet disease relationship to

healthy heart	anaemia)<br>• Heart healthy iron sources<br>• Vegetarian vs non-vegetarian iron sources and how to increase its absorption in the body.
	• Cooking lesson – iron rich healthy lunch
4: All about veggies	• Fresh vs frozen veggies<br>• Why are they important – gut health/fibre, nutrients, chronic disease<br>• Healthy plate model – draw your own plate<br>• Label reading – ingredients (tinned and packaged veggies)
	• Cooking lesson - practice chopping and making veggies yum<br>• Make your own veggies snack or side<br>• Home Challenge: try a veggie you don't usually like, but cooked differently. Take a photo of your dinner plate and compare to healthy plate model.
5: Empowerment	• Careers day – stories from locals<br>• Goal setting<br>• Mapping future pathways<br>• Preparation of empowerment project<br>• Home challenge: ask a family member how they got to be where they are.
6: Healthy bones	• Dietitian to talk on bone health and diet<br>• Adolescents to identify if they are getting enough servings of dairy<br>• Build a goal to meet the recommended dairy intake
	• Cooking lesson - yoghurt and fruit parfait with muesli.
7: Healthy food in the community	• Healthy shopping list<br>• Healthy takeaway options in town<br>• Supermarket plan<br>• Healthy food basket<br>• Saying no to peer pressure<br>• Murri MasterChef and cookbook prep
8: Empowerment project	• Distribution of Big Buddy cookbook<br>• Murri MasterChef competition
